# Development of multiplex gold nanoparticles biosensors for ultrasensitive detection and genotyping of equine herpes viruses

**DOI:** 10.1038/s41598-023-41918-4

**Published:** 2023-09-13

**Authors:** Shimaa M. Ghoniem, Heba E. ElZorkany, Naglaa M. Hagag, Ayman H. El-Deeb, Momtaz A. Shahein, Hussein A. Hussein

**Affiliations:** 1 Department of Virology, Animal Health Research Institute, Agriculture Research Center, Giza, 12618 Egypt; 2 Nanotechnology and Advanced Materials Central Lab, Agriculture Research Center, Giza, 12619 Egypt; 3 Genome Research Unit, Animal Health Research Institute, Agriculture Research Center, Giza, 12618 Egypt; 4 Department of Virology, Faculty of Veterinary Medicine, Cairo University, P.O. Box 12211, Giza, Egypt; 5 Department of Virology, Faculty of Veterinary Medicine, King Salman International University, South Sinai, Egypt

**Keywords:** Microbiology, Molecular biology

## Abstract

Gold nanoparticles (GNPs) biosensors can detect low viral loads and differentiate between viruses types, enabling early diagnosis and effective disease management. In the present study, we developed GNPs biosensors with two different capping agent, citrate-GNPs biosensors and polyvinylpyrrolidone (PVP)-GNPs biosensors for detection of EHV-1 and EHV-4 in multiplex real time PCR (rPCR). Citrate-GNPs and PVP-GNPs biosensors can detect dilution 10^10^ of EHV-1 with mean Cycle threshold (Ct) 11.7 and 9.6, respectively and one copy as limit of detection, while citrate-GNPs and PVP-GNPs biosensors can detect dilution 10^10^ of EHV-4 with mean Ct 10.5 and 9.2, respectively and one copy as limit of detection. These findings were confirmed by testing 87 different clinical samples, 4 more samples were positive with multiplex GNPs biosensors rPCR than multiplex rPCR. Multiplex citrate-GNPs and PVP-GNPs biosensors for EHV-1 and EHV-4 are a significant breakthrough in the diagnosis of these virus types. These biosensors offer high sensitivity and specificity, allowing for the accurate detection of the target viruses at very low concentrations and improve the early detection of EHV-1 and EHV-4, leading to faster control of infected animals to prevent the spread of these viruses.

## Introduction

Equine herpes virus-1 and Equine herpes virus -4 (EHV-1 and EHV-4) are among the most common herpes viruses that affect horses globally^1^. These viruses are known to cause respiratory and neurological illnesses in horses, with EHV-1 being more severe than EHV-4. The symptoms of EHV-1 may include respiratory disease, abortion in female horses, and neurological disease that can lead to paralysis and even death^2^. On the other hand, EHV-4 usually results in milder respiratory symptoms and rarely leads to abortion or neurological illness^3^. Nevertheless, both viruses can have significant economic implications for the equine industry due to their adverse impact on horse health and performance^4,5^.

Detecting and identifying EHV with high sensitivity is crucial for effectively managing and preventing the diseases they cause. A major obstacle in diagnosing these infections is their latent nature, which means that infected animals may not display symptoms or produce antibodies against the virus^6^. Traditional diagnostic methods like virus isolation and serology are often inadequate in detecting the virus in animals with low viral loads or those who are infected but asymptomatic. Therefore, it is essential to develop highly sensitive detection and genotyping techniques to enable early identification, disease monitoring, and control measures to prevent the spread of EHV infections^7^.

Although real-time PCR (rPCR) has been used for EHV detection, it still lacks sensitivity and specificity. Consequently, there is a need for more accurate and efficient diagnostic tools to manage EHV infections. Currently, there is growing interest in developing ultrasensitive detection and genotyping technologies for EHV diagnosis.

These technologies can detect low viral loads and distinguish between different EHV types, providing early diagnosis and effective disease management^8^. Promising diagnostic technologies for EHV detection and genotyping include loop-mediated isothermal amplification (LAMP)^9^, microarray-based assays^10^, and next-generation sequencing (NGS)^11^. These techniques are faster, more sensitive, and have higher resolution than traditional diagnostic methods, making them a valuable tool for the management and prevention of EHV infections in horses.

Gold nanoparticles (GNPs) biosensors are an innovative diagnostic technique that has been created for the ultrasensitive detection and genotyping of viruses. These biosensors rely on the utilization of GNPs as a label to enhance the detection signal of the targeted virus^12^. To fabricate a biosensor, GNPs are coated with nucleic acid probes that have been designed to attach to the specific DNA or RNA sequence of the virus. When the target virus is present, the gold nanoparticles cluster together, resulting in a shift in the plasmon resonance, which can be detected through colorimetry or spectrophotometry^13^.

One of the potential benefits of using GNPs biosensors for virus detection is their high sensitivity and specificity. These biosensors can detect viruses at extremely low concentrations, which is crucial for early detection and accurate diagnosis^14^. In addition, the specificity of the biosensors can be improved by using specific ligands that only bind to the target virus, reducing the risk of false-positive results. Moreover, the use of GNPs as the detection platform allows for easy fabrication, low cost, and high reproducibility, making them ideal for widespread use^15^.

This enables the rapid and accurate detection of the virus, even at low concentrations^16^. GNPs biosensors have exhibited positive outcomes in the detection of various viruses, including Dengue^17^, influenza^18^, Severe acute respiratory syndrome coronavirus 2 (SARS-CoV-2)^19^, and Foot and Mouth Disease (FMD)^20^.

In the present study, we developed GNPs biosensors for detection of EHV-1 and EHV-4 in multiplex rPCR. These biosensors have been validated with the DNA standard of EHV-1 and EHV-4 from the synthetic glycoprotein B (gB) gene of EHV. Application of the GNPs biosensors of EHV-1 and EHV-4 in multiplex rPCR was conducted to test the enhancement effect of these biosensors in the specificity, the analytical sensitivity, dynamic range, efficiency and the detection limit of rPCR.

## Methods

### Synthesis and characterization of GNPs

We have been prepared GNPs using two different capping agent; (Ι) Sodium citrate tribasic dihydrate (Sigma-Aldrich, USA) as reducing and capping agent^21^. Briefly, 1 ml of 1% sodium citrate tribasic dihydrate was added to 10 ml of boiling 1 mM Hydrogen tetrachloroaurate (III) trihydrate (Sigma-Aldrich, USA). (II) Sodium citrate as reducing agent and Polyvinylpyrrolidone (PVP) 10,000 MW (Sigma-Aldrich, USA) as capping agent^22^. Briefly, 5 ml of 1 mM Hydrogen tetrachloroaurate (III) trihydrate was added drop by drop to 30 ml of boiling 1% sodium citrate dihydrate and 0.5 g PVP. The solution's color transformed from pale yellow to deep red, indicating the successful formation of GNPs.

The prepared GNPs were characterized using UV–Vis spectrophotometer, Dynamic light scattering (DLS), zeta potential distribution and high resolution Transmission Electron Microscopy (TEM). The UV–Vis spectra of the prepared GNPs were measured within a wavelength range of 220–800 nm (Varian, Cary 5000). The size distribution of the particles, polydispersity index (PDI) and zeta potential distribution were determined using a Zetasizer Nano-ZS instrument (Malvern Instruments Ltd, UK). Additionally, the morphology of the GNPs and their particle size were examined using TEM (JEOL 2010 F, Japan). The TEM analysis was conducted at an accelerating voltage of 120 kV and the average size of GNPs was obtained through measuring 30–40 particles using TEM image acquisition software and calculating their average size.

### Preparation and characterization of EHV-1 and EHV-4 GNPs biosensors

The gB gene specific primers for EHV-1 and EHV-4 described by Diallo et al.^23,24^, respectively were modified to enable conjugation with GNPs by the addition of the poly (A) spacer and thiol linkers showed in Table 1. The poly (A) tail was used to enhance stability, provide steric hindrance and increase conjugation efficiency of oligonucleotides to GNPs^25,26^. The modified oligonucleotides were deprotected by 0.1 M Dithiothreitol solution (DTT) (Sigma-Aldrich, USA) in the disulfide cleavage buffer then the Nap-5 column (GE Healthcare, UK) was used for desalting and purification of the oligonucleotides.

**Table 1:** Sequences of EHV-1 and EHV-4 gB poly A thiol-linked oligonucleotides.

Viruses	Primers\probes	Sequences (5′–3′)
EHV-1	gB Forward	Thiol group-AAAAAAAAAA-CAT GTC AAC GCA CTC CCA
	gB Reverse	Thiol group-AAAAAAAAAA-GGG TCG GGC GTT TCT GT
	gB Probe	FAM-CCC TAC GCT GCT CC-BHQ1
EHV-4	gB Forward	Thiol group-AAAAAAAAAA-GGGCTATTGGATTACAGCGAGAT
	gB Reverse	Thiol group-AAAAAAAAAA-TAGAATCGGAGGGCGTGAAG
	gB Probe	HEX-CAGCGCCGTAACCAG-BHQ1

The conjugation process of the oligonucleotides with GNPs and the functionalization of GNPs biosensors were carried out as previously described with minor modification^27^; where a 500 nM concentration of thiolated oligonucleotides measured using Qubit™ ssDNA Assay kit (Invitrogen, USA) on Qubit 2.0 fluorometer (Invitrogen, USA) and used for 0.5 mL of GNPs. Phosphate adjustment buffer was added to obtain a final phosphate concentration of 9 mM (Sigma-Aldrich, USA), surfactant solution (10% SDS) (Sigma-Aldrich, USA) was added to enhance the stability of the system and prevent the aggregation of GNPs at high salt concentrations. Also, salting buffer was added to decrease the electrostatic repulsion between negatively charged citrates and oligonucleotides, resulting in a shorter distance between the GNPs and oligonucleotides^28^. The total required salting buffer was divided into eight equal additions to achieve a final concentration of 0.3 M Sodium chloride (Sigma-Aldrich, USA) and added over a course of two days. After the final addition of salt, the particles were left to equilibrate overnight.

The EHV-1 and EHV-4 biosensors were characterized using UV–Vis spectrophotometer, DLS, TEM and agarose gel electrophoresis^29^. 2% agarose (Sigma-Aldrich, USA) was prepared in 0.5% Tris–acetate-EDTA (TAE) buffer (Thermo Fisher Scientific, USA) then 10 μL of sample was mixed with 2 μL glycerol (Sigma-Aldrich, USA) and loaded into agarose gel. The electrophoresis apparatus (Cleaver, UK) was adjusted to 90 V for 30 min.

### Multiplex rPCR assay

Multiplex rPCR assay was conducted using Quantifast Multiplex PCR kit (Qiagen, Germany) with a thermal profile according to the manufacturer’s instructions in Gentier 48R real-time PCR system (Tianlong, China). EHV-1 and EHV-4 gB synthesized gene (Biobasic, Canada) were serially diluted, and dilutions from10^10^ to 10^0^ were amplified in triplicate for the generation of standard curves. The R squared values (Rsq) and the detection limit of the multiplex assay were determined^20^.

### Evaluation of specificity and sensitivity for EHV-1 and EHV-4 multiplex GNPs biosensors with rPCR

The EHV-1 and EHV-4 multiplex GNPs biosensors were evaluated for the analytical specificity using EHV-1 and EHV-4 gB synthetized genes. *Streptococcus equi bacteria (S.equi)*, which cause respiratory manifestations similar to EHV infection in equine species, was obtained from Equine Bacteriology Disease Unit, Animal Health Research Institute, purified viral DNA for EHV-2 and EHV-5 were obtained from Institute of Virology, Freie Universität, Berlin used as negative control. Different GNPs biosensors concentrations (300, 400, 500, 600 nM) were used to generate standard curves and determine their detection limit as explained in the previous step.

### Application of the EHV1and EHV-4 multiplex GNPs biosensors rPCR on clinical sample

Eighty seven different clinical samples (nasal swabs, vaginal swab, aborted fetus, lung, liver and spleen of dead foals and adult horses) were collected in February 2019 to December 2022 in Egypt. DNA in samples was extracted using QIAamp DNA mini kit (Qiagen, Germany) and tested using multiplex rPCR, multiplex Citrate-GNPs biosensors rPCR and multiplex PVP-GNPs biosensors rPCR.

## Results

### Synthesis and characterization of GNPs

**Figure 1:**
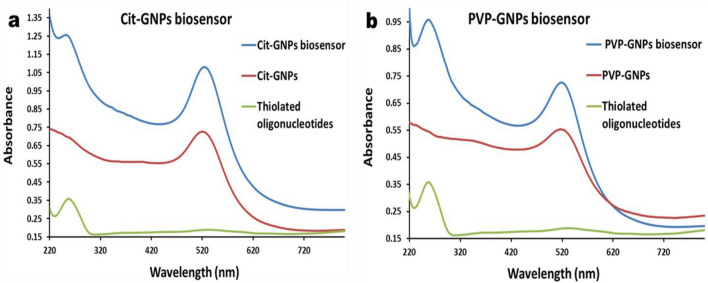
UV–Vis spectrophotometer of the GNPs and GNPs biosensors: (**a**) Citrate-GNPs absorption peak at 520 nm (red curve), free thiolated oligonucleotides absorption peak at 260 nm (green curve), citrate-GNPs biosensor, two absorption peaks one for citrate-GNPs at 520 nm and another one for the conjugated thiolated oligonucleotides at 260 nm (blue curve). (**b**) PVP-GNPs absorption peak at 517 nm (red curve), free thiolated oligonucleotides absorption peak at 260 nm (green curve), PVP-GNPs biosensor, two absorption peaks one for PVP-GNPs at 517 nm and another one for the conjugated thiolated oligonucleotides at 260 nm (blue curve).

**Figure 2:**
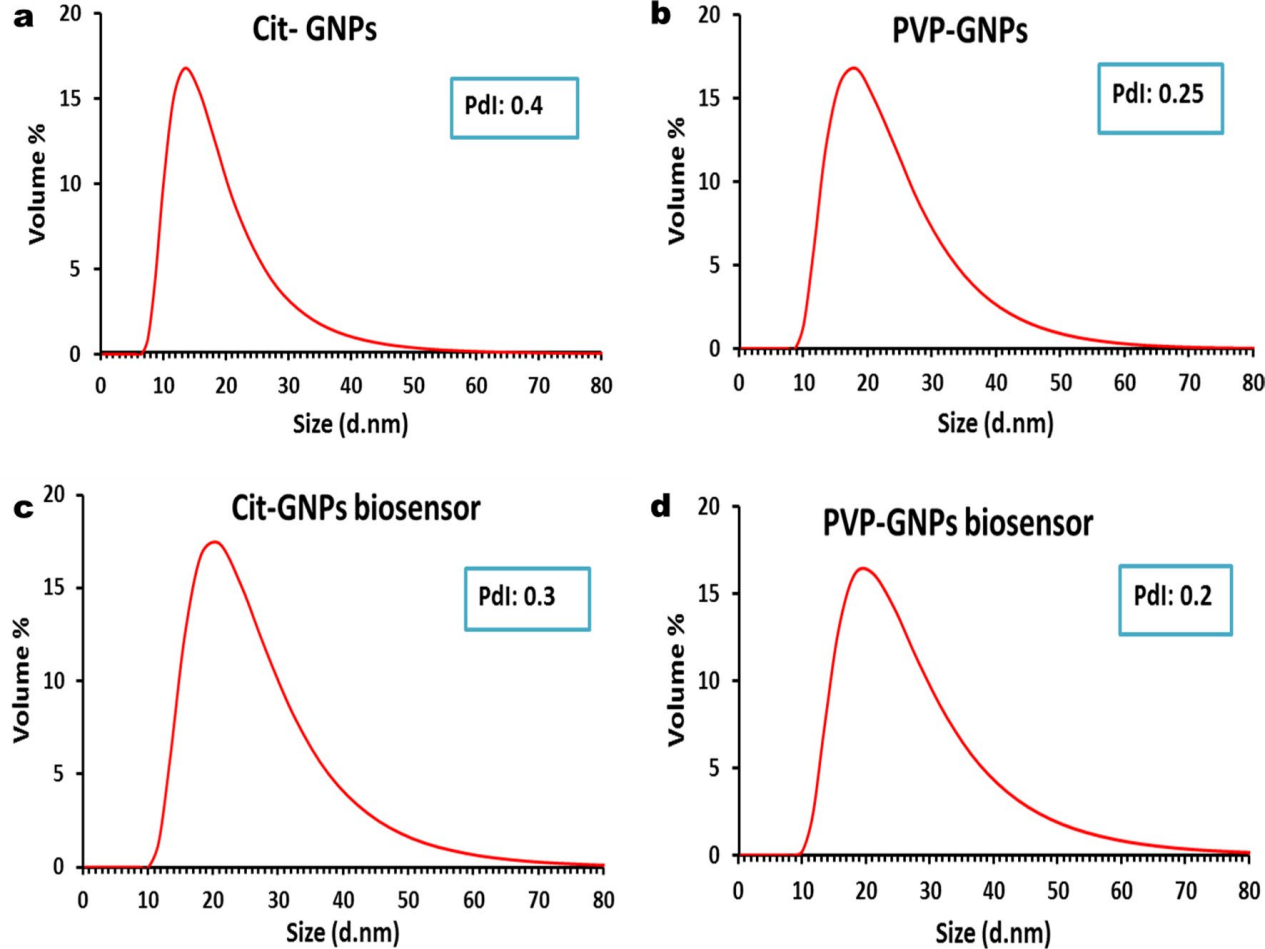
The dynamic light scattering of the GNPs: (**a**) The curve demonstrates a peak of size distribution by volume representing citrate-GNPs with an average hydrodynamic diameter of 17 nm and PDI of 0.4. (**b**) Curve demonstrates a peak of size distribution by volume representing PVP-GNPs with an average hydrodynamic diameter of 21nm and PDI of 0.25. (**c**) Curve demonstrates a peak of size distribution by volume of citrate-GNPs biosensor showing slight increase in average hydrodynamic diameter of 24 nm due to the conjugation with PDI of 0.3. (**d**) Curve demonstrates a peak of size distribution by volume of PVP-GNPs biosensor showing slight increase in average hydrodynamic diameter of 24 nm due to the conjugation with PDI of 0.2.

**Figure 3:**
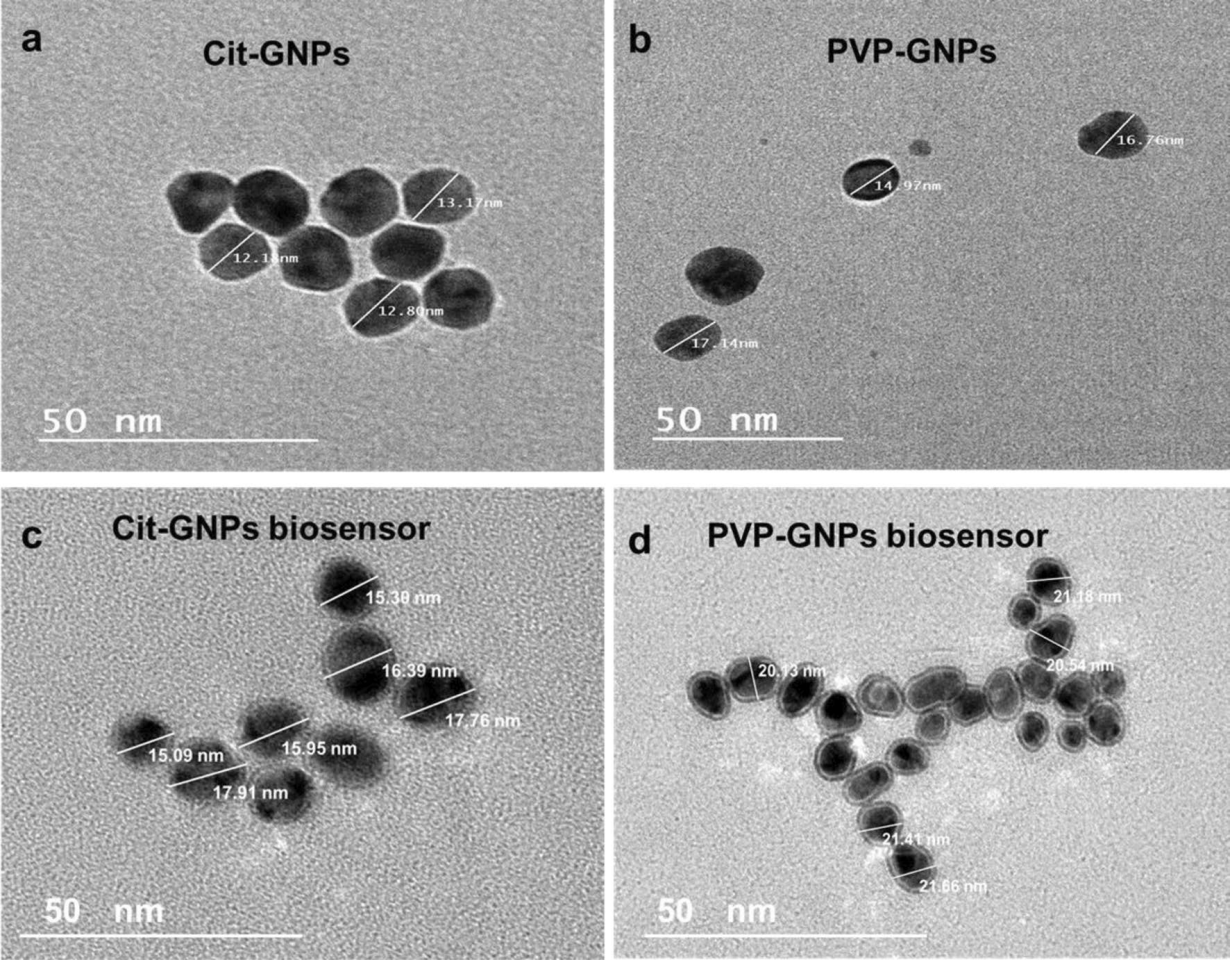
GNPs under the transmission electron microscope: (**a**) The electron microscopy image shows 13 ± 1 nm citrate-GNPs. (**b**) The electron microscopy image shows 15 ± 3 nm PVP -GNPs. (**c**) The electron microscopy image reveals slight increase in citrate –GNPs with an average size 16 nm and formation of grey zone of oligonucleotides conjugation. (**d**) The electron microscopy image reveals slight increase in PVP –GNPs with an average size 21 nm and formation of grey zone of oligonucleotides conjugation.

The UV–vis absorption spectrum of the prepared citrate-GNP showed an absorption maximum at 520 nm (Red curve) (Fig. 1a), PVP-GNPs showed an absorption maximum at 517 nm (Red curve) (Fig. 1b). According to DLS, the size distribution of citrate-GNPs exhibits a peak at approximately 17 nm with polydispersity index (PDI) value 0.4 (Fig. 2a), the size distribution of PVP–GNPs exhibit a peak at approximately 21 nm with PDI value 0.25 (Fig. 2b). Zeta potential distribution showed a peak of -38.3 mV for citrate-GNPs and -8.5 mV for PVP-GNPs. As a final point, the characteristics of the synthesized GNPs were further confirmed by TEM imaging where the synthesized citrate-GNPs were 13 ± 1 nm in diameter (Fig. 3a), while the PVP-GNPs were 15 ± 3 (Fig. 3b).

### Preparation and characterization of EHV-1 and EHV-4 GNPs biosensors

EHV-1 and 4 GNPs biosensors were prepared through conjugation of thiolated oligonucleotides which targeted EHV gB gene to citrate-GNPs and PVP-GNPs. The conjugation of the oligonucleotides to GNPs was checked by different characterization methods. By visual inspection, there was no difference in color between conjugated and unmodified GNPs, which indicates that they are well-functionalized with no visible aggregates (Supplementary Fig. 1). UV–Vis spectrophotometer absorbance of the citrate-GNPs biosensors shown in (Fig. 1a), absorption peak of 260 nm was observed with thiolated oligonucleotides (Green curve). Whereas, the citrate-GNPs biosensor showed two absorption peaks (representing the conjugation of thiolated oligonucleotides to citrate-GNPs), one for the citrate-GNPs at 520 nm and second for the thiolated oligonucleotides at 260 nm (Blue curve). UV–Vis spectrophotometer absorbance of the PVP-GNPs biosensors shown in (Fig. 1b), thiolated oligonucleotides absorption peak was at 260 nm (Green curve). On the other hand, the PVP-GNPs biosensor showed two absorption peaks, (representing the conjugation of thiolated oligonucleotides to PVP-GNPs), PVP-GNPs Absorption peak at 517 nm and the thiolated oligonucleotides absorption peak at 260 nm (Blue curve). Furthermore, the preparation of GNPs biosensors was analyzed using DLS analysis, as the conjugation of EHV thiolated oligonucleotides cause size enlargement that shows peak shift of the hydrodynamic size of the GNPs from 17 to 24 nm for citrate-GNPs (Fig. 2c), and from 21 to 24 nm for PVP-GNPs (Fig. 2d). The TEM images revealed a slight increase in GNPs size following EHV thiolated oligonucleotides conjugation, the average size of citrate-GNPs increased to 16 nm (Fig. 3c), and of PVP-GNPs increased to 21 nm (Fig. 3d). Moreover, a distinct grey zone developed around the GNPs conjugated with oligonucleotides that indicating successful conjugation.

**Figure 4:**
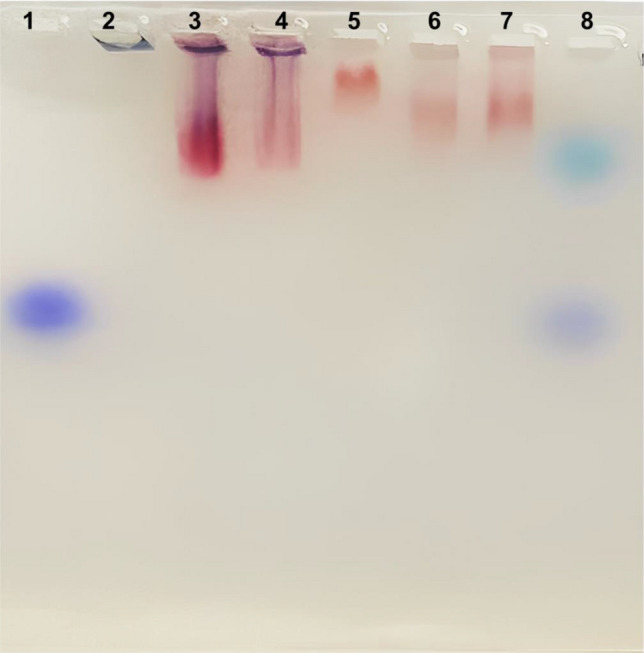
Agarose gel analysis (cropped image) of synthetized GNPs and prepared GNPs biosensors. (Lane 1) Marker; (Lane 2) Unfunctionalized citrate-GNPs did not migrate in the gel; (lane 5) PVP-GNPs slightly migrate as a result of PVP capping. (Lane 3 and 4) two different concentrations of citrate-GNPs biosensors and (lane 6 and 7) two different concentrations of PVP-GNPs biosensors migration are visible under natural light. Original gel is presented in Supplementary Fig. 5.

Finally, agarose gel electrophoresis (Fig. 4) showed the negative electrokinetic potential of prepared citrate-GNPs biosensors (lane 3 and 4 represent two different concentrations of the biosensor) and PVP-GNPs biosensors (lane 6 and 7 represent two different concentrations of the biosensor) that observed with their electrophoretic mobility confirming the success preparation of EHV-GNPs biosensors. However, unfunctionalized citrate-GNPs (lane 2) did not migrate in the gel, PVP-GNPs (lane 5) slightly migrate as a result of PVP capping.

Indeed, all these findings ensure that we successfully prepared citrate-GNPs and PVP-GNPs biosensors to EHV1 and 4 that can be used in detection and differentiation of EHV-1 and 4.

### Multiplex rPCR assay

To determine the enhancement detection of EHV-1 and 4 GNPs biosensors, first we made ten-fold serial dilution of the EHV-1 and EHV-4 gB synthetic gene and used dilutions from 10^10^ to 10^0^ (equal 1 copy) to make multiplex rPCR standard curves in triplicate and get the mean Cycle threshold (Ct) to determine its detection limit. Multiplex rPCR assay can detect 10^10^ dilution of EHV-1with mean Ct 13.8 while EHV-4 at 13.1, and the detection limit was 10^2^ (equal 100 copies) for both EHV-1 and EHV-4 (Supplementary Fig. 2a-b, Table 2).

**Table 2:** Results of analytical sensitivity and detection limit of multiplex rPCR, multiplex citrate-GNPs and multiplex PVP-GNPs biosensors.

	Multiplex rPCR	Multiplex citrate-GNPs biosensors	Multiplex PVP-GNPs biosensors
Mean Ct dilution 10^10^ EHV-1	13.8	11.7	9.6
Mean Ct dilution 10^2^ EHV-1	32.8	29.9	28.8
Mean Ct dilution 10^1^ EHV-1	Not detected	32.3	31.5
Mean Ct dilution 10^0^ EHV-1	Not detected	34.9	34.1
Detection limit for EHV-1	100 copies	1 copy	1 copy
Mean Ct dilution 10^10^ EHV-4	13.1	10.5	9.2
Mean Ct dilution 10^2^ EHV-4	32.3	30.5	29.5
Mean Ct dilution 10^1^ EHV-4	Not detected	31.9	31.3
Mean Ct dilution 10^0^ EHV-4	Not detected	34.7	34.2
Detection limit for EHV-4	100 copies	1 copy	1 copy

### Evaluation of specificity and sensitivity for EHV-1 and EHV-4 multiplex GNPs biosensors with rPCR

Specificity of the EHV-1 and EHV-4 GNPs biosensors was evaluated using EHV-1 and EHV-4 gB synthetic genes and *S.equi*, EHV-2 and EHV-5 DNA as negative control. Both GNPs biosensors amplified only the specific gB genes with no cross amplification in the multiplex assay. *S.equi* was amplified by its specific primers and probe and did not amplified either by citrate-GNPs or PVP-GNPs biosensors (Supplementary Fig. 3). EHV-2 and EHV-5 DNA did not amplified either by citrate-GNPs or PVP-GNPs biosensors (Supplementary Fig. 4).

**Figure 5:**
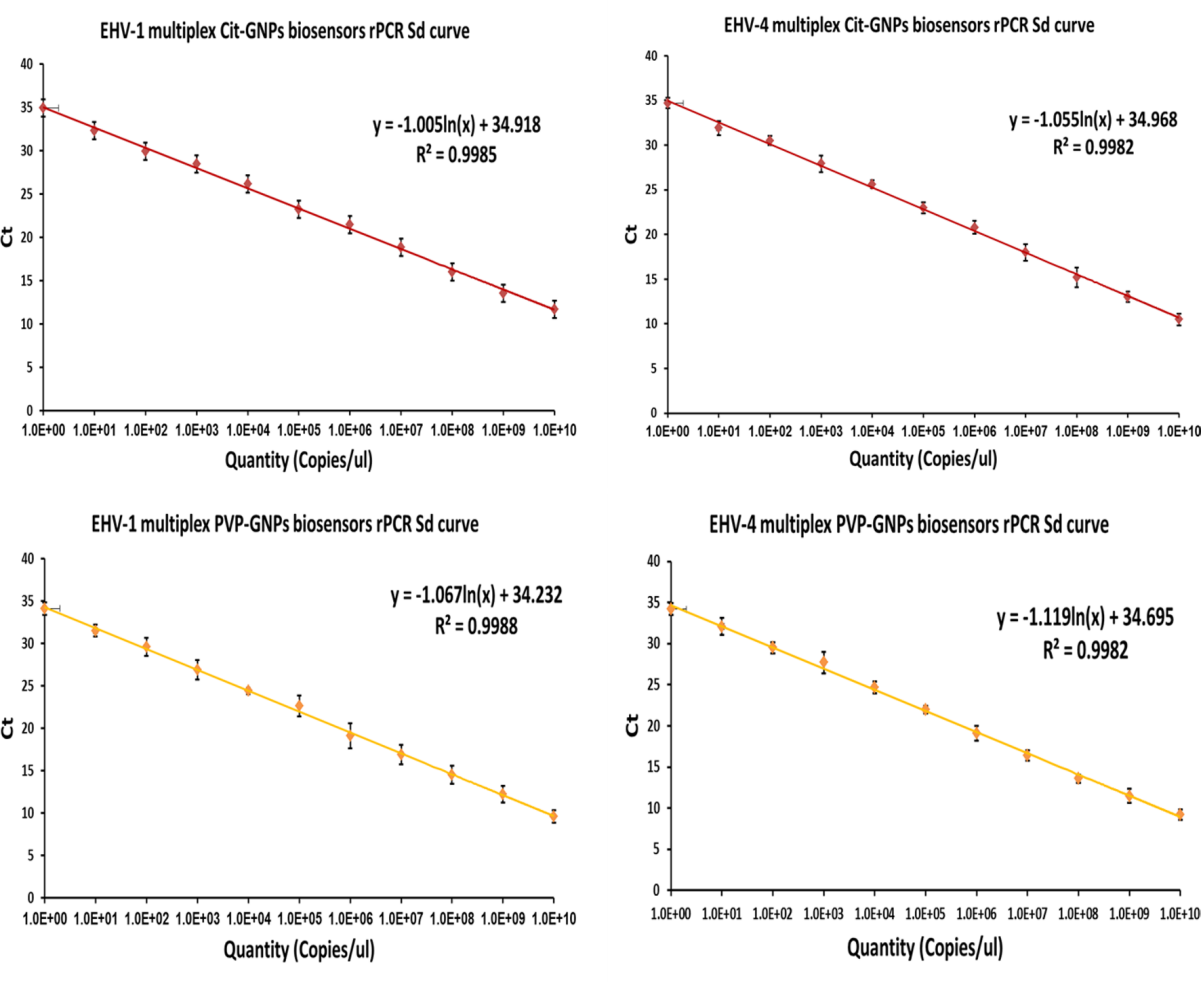
Standard curve of EHV-1 and EHV-4 multiplex rPCR assay using GNPs biosensors. (**a**) Standard curve of EHV-1 multiplex rPCR assay using EHV-1 and 4 citrate-GNPs biosensor shows its detection limit (1 copy). (**b**) Standard curve of EHV-4 multiplex rPCR assay using EHV1 and 4 citrate-GNPs biosensor shows its detection limit (1 copy). (**c**) Standard curve of EHV-1 multiplex rPCR assay using EHV1 and 4 PVP-GNPs biosensor shows its detection limit (1 copy). (**d**) Standard curve of EHV-4 multiplex rPCR assay using EHV1 and 4 PVP-GNPs biosensor shows its detection limit (1 copy). Amplification curves in Supplementary Fig. 7.

To recognize the optimum GNPs biosensors concentration, we tested four dilutions (300, 400, 500, 600 nM) in rPCR and found that GNPs biosensors concentration of 500 nM had the most acceptable results as this concentration of citrate-GNPs and PVP-GNPs biosensors can detect dilution 10^10^ of EHV-1 with mean Ct 11.7 and 9.6, respectively and one copy as limit of detection (Table 2, Fig. 5a–c) , while this concentration of citrate-GNPs and PVP-GNPs biosensors can detect dilution 10^10^ of EHV-4 with mean CT 10.5 and 9.2, respectively and one copy as limit of detection (Table 2, Fig. 5b–d).

### Application of the EHV1and EHV-4 multiplex GNPs biosensors rPCR on clinical samples

Eighty seven clinical samples were tested using multiplex rPCR, multiplex citrate-GNPs biosensors rPCR and multiplex PVP-GNPs biosensors rPCR. The interpretation of the results was based on the Ct values. If the Ct values were 35 or less, the test result was considered positive. If the Ct values fell between 36 and 40, the results were considered questionable and required a retest using an alternative method. Finally, if the Ct values were above 40 and equal to zero, the result was negative. Out of 87 samples, 29 samples were positive for detection of EHV DNA using multiplex rPCR. EHV-1 DNA were detected in 11 of 20 nasal swabs, one of 9 Liver samples, 2 of 14 spleen samples, 4 of 36 lung samples, 2 of 7 aborted fetuses and the vaginal sample. EHV-4 DNA was detected in one of 14 spleen samples and 7 of 36 lung samples. Using multiplex citrate-GNPs biosensors rPCR and multiplex PVP-GNPs biosensors rPCR, EHV DNAs were detected in 33 of 87. EHV-1 DNA were detected in 15 of 20 nasal swabs, one of 9 Liver samples, 2 of 14 spleen samples, 4 of 36 lung samples, 2 of 7 aborted fetuses and the vaginal sample. EHV-4 DNA was detected in one of 14 spleen samples and 7 of 36 lung samples (Table 3). Ct values were used to analyze the difference in detection levels of EHV-1 and 4 DNA between multiplex rPCR, multiplex citrate-GNPs biosensors rPCR and multiplex PVP-GNPs biosensors rPCR. There was a significant enhancement in EHV-1 and EHV-4 DNA detection in all 29 positive samples detected by multiplex rPCR (Supplementary Dataset File). There were 4 nasal swabs samples their Ct values for EHV-1 DNA detection with multiplex rPCR were more than 35, so according to this assay those samples need to retest with different technique. While the same samples when tested with multiplex citrate-GNPs biosensors rPCR and multiplex PVP-GNPs biosensors rPCR, their Ct values were less than 35 so those samples were considered positive.

**Table 3:** Results of multiplx rPCR and multiplex GNPs biosensors rPCR for detection of EHV-1 and EHV-4 in collected clinical samples.

Sample type	Total Sample No	Multiplex rPCR Positive sample No	EHV-1	EHV-4	Multiplex GNPs biosensors rPCR Positive sample No	EHV-1	EHV-4
Nasal Swabs	20	11	11	–	15	15	–
Liver	9	1	1	–	1	1	–
Spleen	14	3	2	1	3	2	1
Vaginal swab	1	1	1	–	1	1	–
Lung	36	11	4	7	11	4	7
Aborted fetus	7	2	2	–	2	2	–

These results confirm that the multiplex GNPs biosensors rPCR for EHV-1 and 4 are more sensitive and specific for the detection of EHV-1 and 4 than multiplex rPCR.

## Discussion

Control of EHV incurs expenses for diagnostic testing, vaccination, and biosecurity measures^30^. The economic impact of EHV on the equine industry is considerable as it results in reduced performance and prolonged recovery periods for affected horses^31^. The development of ultrasensitive detection techniques for EHV such as GNPs biosensors is of great importance and plays a major role on the success of effective disease control strategies.

In the present study, we developed two multiplex GNPs biosensors with different capping agent (citrate-PVP) for detection of EHV-1 and 4. The choice of capping agent affects the size, shape, and stability of the nanoparticles, as well as their interactions with biological molecules^32^. Common capping agents for GNPs include thiol-containing molecules (citrate, glutathione, and cysteine) make strong covalent bonds with the gold surface^33^. Other capping agents include polymers, surfactants, and proteins, provide steric hindrance and electrostatic stabilization. The type and concentration of the capping agent used affect the surface charge and hydrophobicity of the GNPs, which can impact their interactions with targeted molecules^34,35^.

Parameters used to characterized the prepared GNPs (citrate-GNPs: absorption maximum 520 nm, size distribution 17 nm with PDI values 0.4 and size 13 ± 1 nm, PVP-GNPs: absorption maximum 517 nm, size distribution 21 nm with PDI value 0.25 and size15 ± 3 nm) indicate that the PVP-GNPs were slight bigger in size than the citrate-GNPs and this is may be a result of capping the GNPs with PVP which make them bigger and more stable.

The difference in measured size between the DLS and TEM comes from the difference in nature of each measurement technique, whereas TEM depends on electron beam that gives a high resolution image which in return can be used to determine the particle’s diameter, DLS depends on laser beam that scatters on the surface of nanoparticles and by using the intensity of the scattered laser the system gives estimation of the hydrodynamic diameter of the particle in the solution which is slightly larger than the real diameter^36^.

The conservative region in the gB gene of EHV^23,24^ was targeted during preparation of EHV-1 and EHV-4 GNPs biosensors utilizing the protocol previously published^27^. The obtained same color of the biosensors after conjugation compared with un-modified GNPs without visible aggregation confirming that they were well-functionalized GNPs biosensors^27^.

The changes in the size of GNPs (size distribution peak became 24 nm instead of 17 nm and 21 nm for citrate-GNPs and PVP-GNPs, respectively and particles average size in TEM images became 16 nm and 21 nm for citrate-GNPs and PVP-GNPs, respectively) referred to binding of thiolated oligonucleotides to GNPs surface^37^. This conjugation is believed to be thiol linking. GNPs-thiol bonding occurs through GNPs-S interaction or thiolated DNA. In GNPs-S interaction, thiols directly react with oxidized gold surfaces, forming GNPs-S bonds through oxidation–reduction reactions. This process reduces gold oxide and allows thiols to adsorb onto the reduced gold surface, forming gold (I) thiolates. Thiolated DNA, with a thiol group at the end, is commonly used to modify GNPs due to the strong gold-sulfur affinity. Commercial thiolated single-stranded DNAs often have protective disulfide bonds, like mercaptopropanol (for the 3'-terminal) or mercaptohexanol (MCH) (for the 5'-terminal). Prior to conjugating with thiolated DNAs, the disulfide bond must be converted into two thiol groups using reducing agents like dithiothreitol (DTT) or tris(2-carboxyethyl) phosphine hydrochloride (TCEP-HCl)^38^.

Thiolated oligonucleotides conjugation to GNPs can be analyzed by the electrophoretic mobility assay^29^. The migration difference in agarose gel electrophoresis between citrate-GNPs and the conjugated biosensor is due to the charge of the particles. Citrate-GNPs, with their negatively charged surface, experience strong repulsion from the negatively charged agarose gel matrix, hindering their migration within the gel. However, when thiolated DNA is conjugated to the GNPs reduce the repulsive forces and enabling freer migration through the gel matrix. The migration of PVP-GNPs in agarose gel electrophoresis is facilitated by their reduced surface charge, which weakens the repulsion between the GNPs and the negatively charged gel matrix. This reduced repulsion enables easier migration of the GNPs through the gel. The presence of the PVP capping agent further enhances their migration by providing steric hindrance and electrostatic stabilization, preventing aggregation and maintaining stability. The protective layer formed by the polymer allows the individual GNPs to migrate through the gel without significant hindrance^39,40^.

EHV-1 and EHV-4 biosensors specificity were tested and they showed high specificity to their targeted gB gene without any cross amplification and did not amplify *S.equi*, EHV-2, or EHV-5. To determine the optimum GNPs biosensors concentration that reveals the lowest Ct value for 10^10^ dilution and lowest detection limit, rPCR was carried out with different GNPs biosensors concentrations, and results revealed that 500 nM was the optimum concentration from citrate-GNPs biosensors which enhance the EHV-1 detection in 10^10^ dilution by 2.1 cycles and from PVP-GNPs biosensors by 4.2 cycles. Also, this concentration from citrate-GNPs biosensors enhance the EHV-4 detection in 10^10^ dilution by 2.6 cycles and from PVP-GNPs biosensors by 3.9 cycles. These results clearly demonstrated that the capping GNPs with PVP made the GNPs more stable and enhance their efficiency than the citrate capped GNPs.

The significant improvement in detection limit for both biosensors was found to be as low as one copy, compared to rPCR which was with a detection limit of 100 copies. The causes that the EHV-GNPs biosensors enhance the detection limit of rPCR could be due to: (a) GNPs enhance PCR amplification by reducing primer melting temperatures and increasing its specificity. (b) They adsorb polymerases, boosting PCR efficiency. (c) GNPs also adsorb PCR products, aiding detachment during denaturing. (d) They disperse heat well in shortened cycles. (e) GNPs modulate DNA polymerase activity, mimicking hot start effect. Given their biocompatibility, large surface area, ease of preparation, and the possibility of surface modification, GNPs offer a valuable tool for boosting the PCR amplification process^41,42^.

GNPs-assisted PCR was developed for detection of EHV-1. Unmodified citrate-GNPs were added to PCR mixture as PCR additive to enhance the overall amplification process. This method increase PCR amplification yield with a detection limit of 10^2^ EHV-1DNA copies compared to conventional PCR, which had a detection limit of 10^5^ to 10^4^ DNA copies^43^. On the other hand, EHV multiplex GNPs biosensors rPCR in our study can detect and genotyping EHV-1 and EHV-4 with detection limit one copy of EHV-1 and EHV-4 DNA compared to multiplex rPCR which had a detection limit of 10^2^ DNA copies. This high sensitivity makes them suitable for detection of EHV infection in animals during the early stage, even before the onset of clinical signs and also in detecting the latent infected animals reflecting on the disease monitoring and control to prevent the spread of EHV infections^44^.

Application of EHV-1 and EHV-4 multiplex citrate-GNPs and PVP-GNPs biosensors rPCR in clinical samples (n = 87), resulted in detection of EHV-1 and EHV-4 DNA in more samples (33 samples) compared to multiplex rPCR, which detected only 29 samples. These findings resulted from that multiplex citrate-GNPs and PVP-GNPs biosensors rPCR assays detected four more positive samples for EHV-1 out of the 87 samples tested, compared to the multiplex rPCR. Low amount of virus present in the nasal swabs collected from adult horse may be the reason, as their Ct values were 35.3, 36.8, 35.6 and 36.1 in multiplex rPCR and consider borderline values. Additional testing is required in such samples leading to delay in releasing results and increase the costs associated with the testing process, as it requires more time and resources to perform. While the same samples were considered positive in multiplex citrate-GNPs and PVP-GNPs biosensors rPCR assays, as using GNPs biosensors enhance their Ct values to be 32.8, 34.6, 33.2 and 33.6 in multiplex citrate-GNPs biosensors rPCR, and 32.6, 33.5, 31.8 and 32.2 in multiplex PVP-GNPs biosensors rPCR.

## Conclusion

It is crucial to enhance the efficiency of diagnostic tools available for EHV-1and EHV-4 in developing countries. In the present study, the use of EHV-1 and EHV-4 multiplex citrate-GNPs or multiplex PVP-GNPs biosensors demonstrated their greater analytical and clinical performance for the EHV-1 and EHV-4 diagnosis. These biosensors offer a remarkable capability to detect even a single copy of viral DNA, ensuring highly sensitive detection during early and latent infection of EHV. Moreover, the multiplex nature of these biosensors allows for simultaneous detection and differentiation of both EHV-1 and EHV-4, facilitating efficient and comprehensive diagnostic processes. By enabling early and accurate detection, the use of multiplex GNPs biosensors rPCR greatly contributes to effective disease management, minimizing the impact of EHV on the equine population. To our knowledge, this study is the first to report the use of PVP-GNPs biosensors for detection and genotyping of EHV types 1 and 4.

### Supplementary Information

Supplementary Figures.

Supplementary Dataset File.

## Data Availability

The datasets used and/or analyzed during the current study are available from the corresponding author on reasonable request.
