# Supplementary material for: Development of multiplex gold nanoparticles biosensors for ultrasensitive detection and genotyping of equine herpes viruses
Source: Sci Rep. 2023 Sep 13;13:15140. doi: 10.1038/s41598-023-41918-4 (PMC10500010; doi:10.1038/s41598-023-41918-4)
Supplement: Supplementary file 1 — Supplementary Figures. [file 41598_2023_41918_MOESM1_ESM.docx]

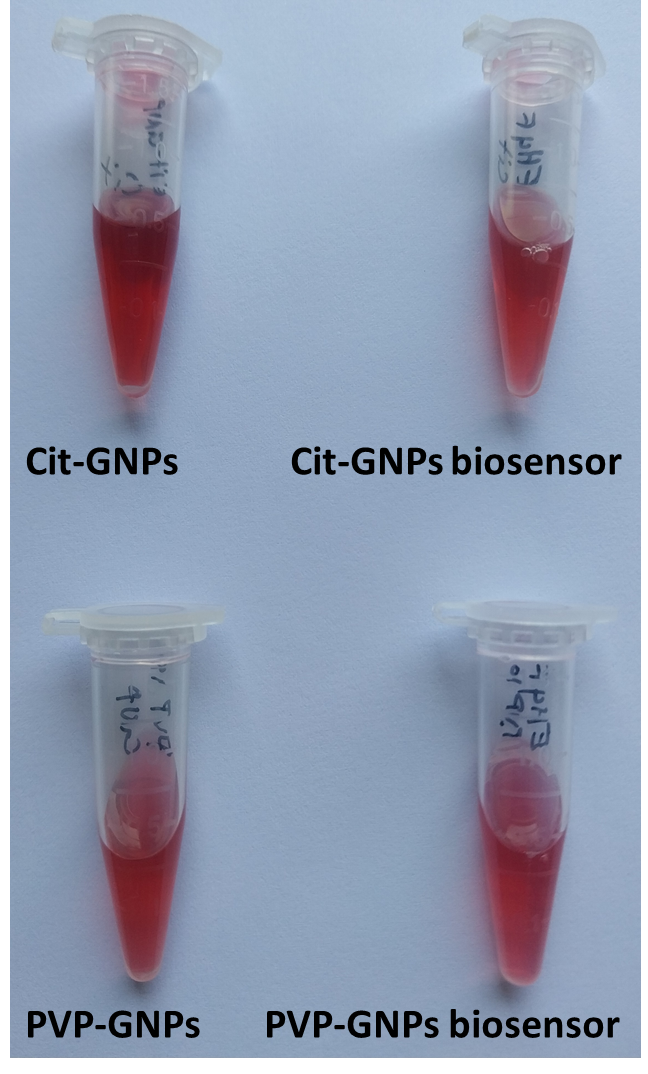


**Supplementary Figure 1.** Visual inspection for unmodified GNPs and Prepared GNPs biosensors. There was no difference in color between unmodified GNPs and both Citrated-GNPs and PVP-GNPs biosensors, which indicates that they are well-functionalized with no visible aggregates.

**
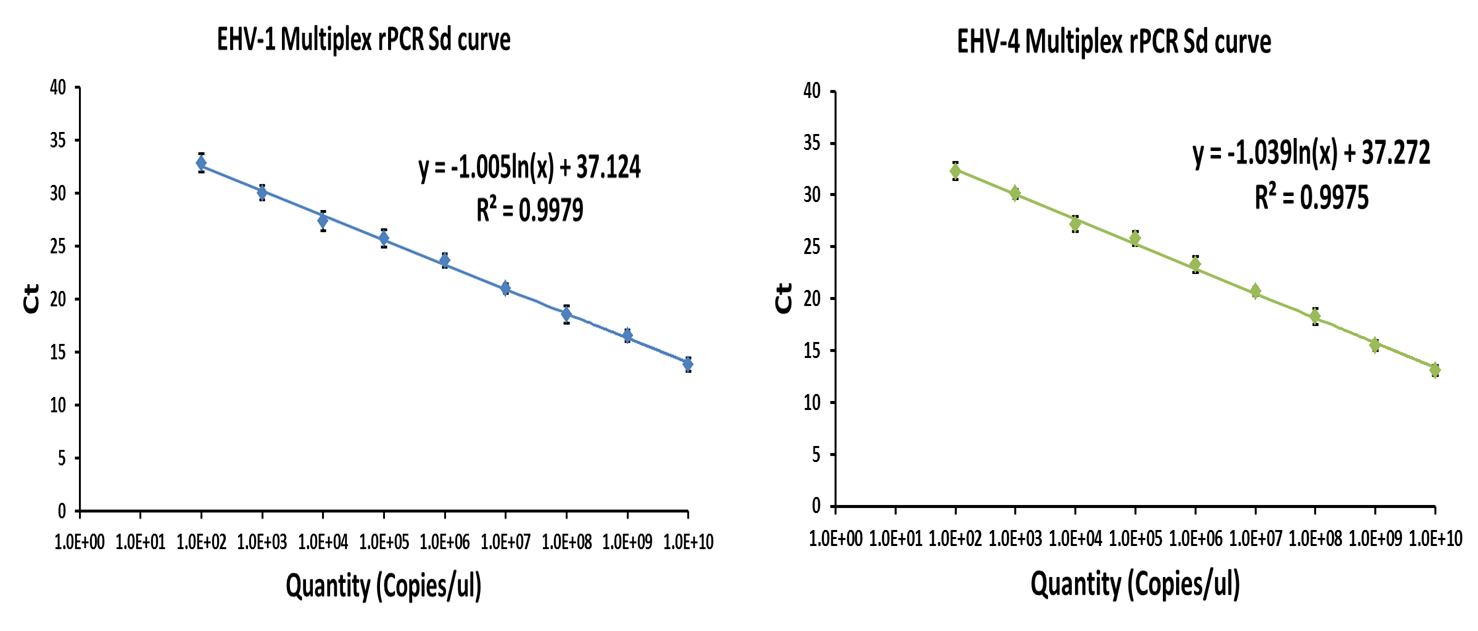
**

**Supplementary Figure 2.** Standard curves of EHV-1 and 4 multiplex rPCR assays. (a) Standard curve of EHV-1 in the multiplex rPCR assay showed its detection limit (100 copies). (b) Standard curve of EHV-4 in the multiplex rPCR assay showed its detection limit (100 copies).


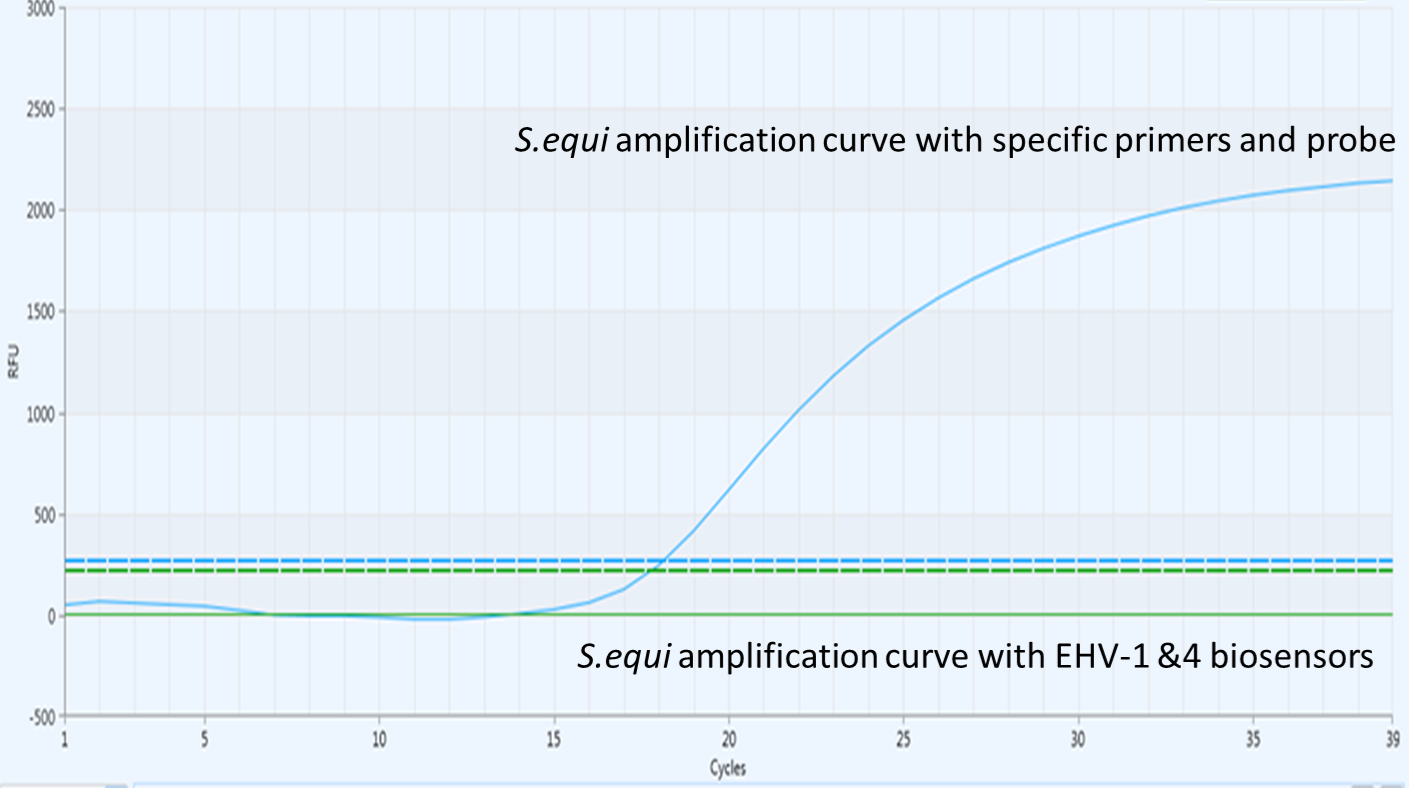


**Supplementary Figure 3.** Amplification curve of *S.equi* by its specific primers and probe, citrate- GNPs and PVP- GNPs biosensors. *S.equi* was amplified by its specific primers and probe (blue amplification curve) and did not amplified either by citrate- GNPs or PVP- GNPs biosensors (blue and green lines).

**
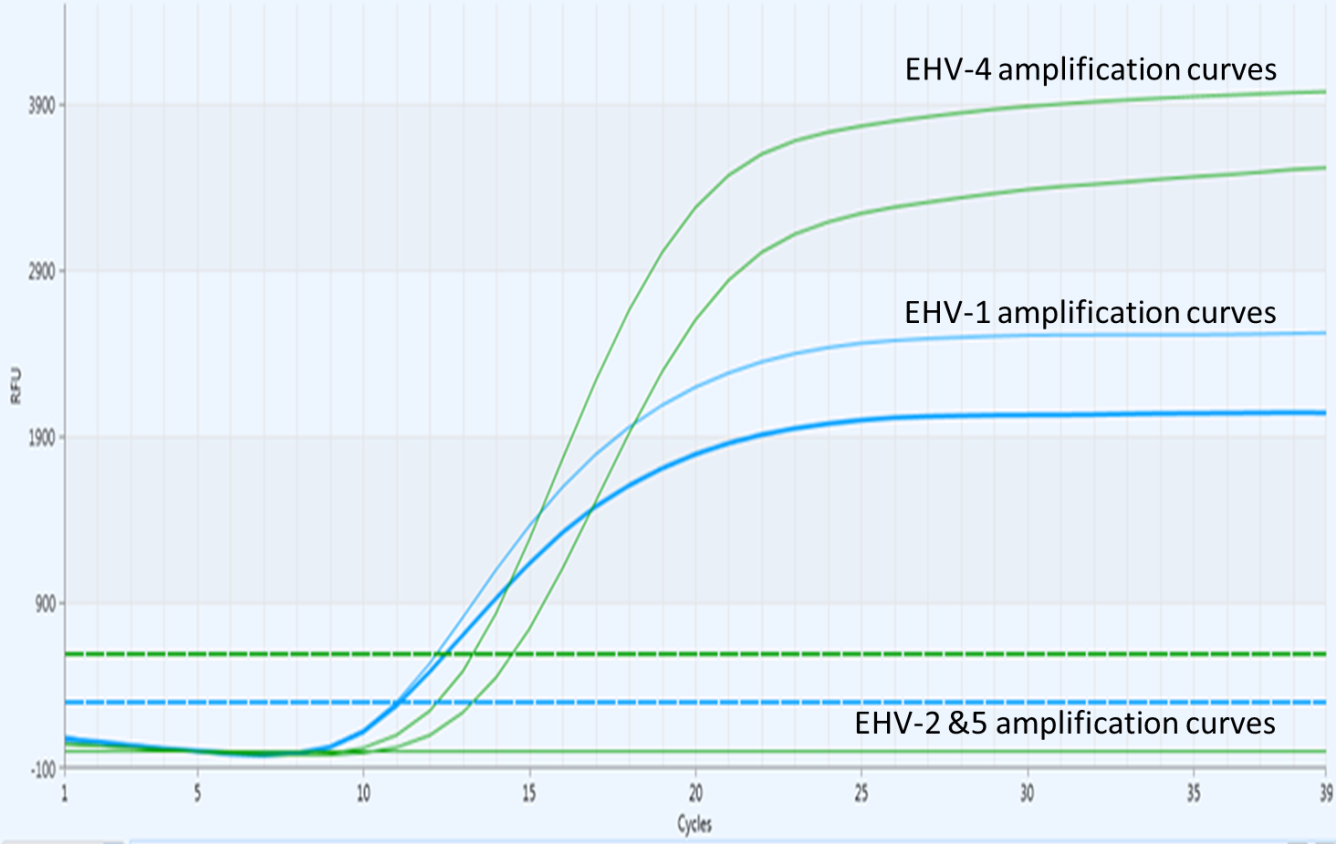
**

**Supplementary Figure 4.** Amplification curves of EHV-2 & EHV-5 with EHV-1 and EHV-4 multiplex rPCR citrate- GNPs and PVP- GNPs biosensors. EHV-1 and EHV-4 multiplex rPCR citrate- GNPs and PVP- GNPs biosensors amplified EHV-1 DNA (blue amplification curves) and EHV-4 DNA (green amplification curves) and did not amplified either EHV-2 or EHV-5 DNA (blue and green lines).

**
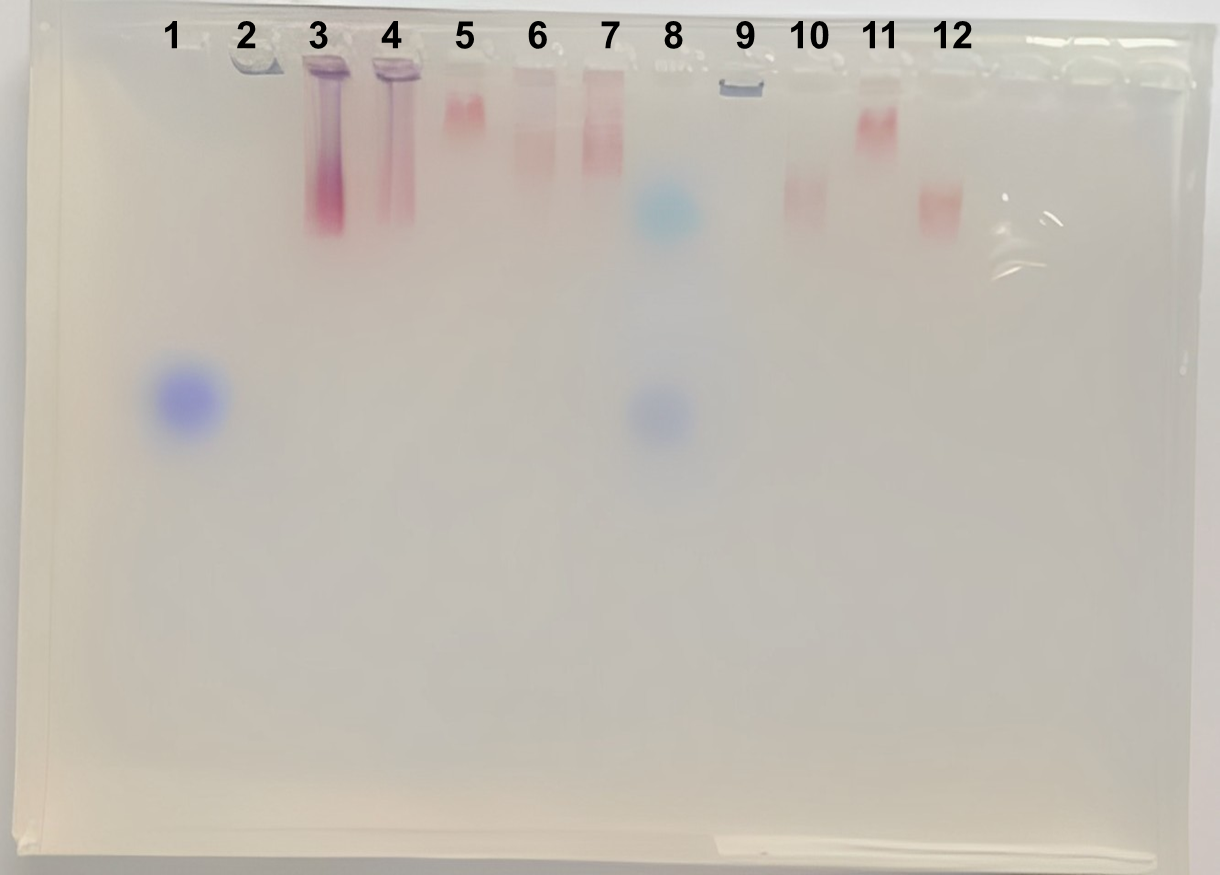
**

**Supplementary Figure 5.** Agarose gel analysis (uncropped image) of synthetized GNPs and prepared GNPs biosensors. (Lane 1) Marker; (Lane 2 and 9) Unfunctionalized citrate- GNPs did not migrate in the gel; (lane 5 and 11) PVP- GNPs slightly migrate as a result of PVP capping. (Lane 3, 4 and 10) different concentrations of citrate- GNPs biosensors and (lane 6, 7 and 12) two different concentrations of PVP- GNPs biosensors migration are visible under natural light.

**
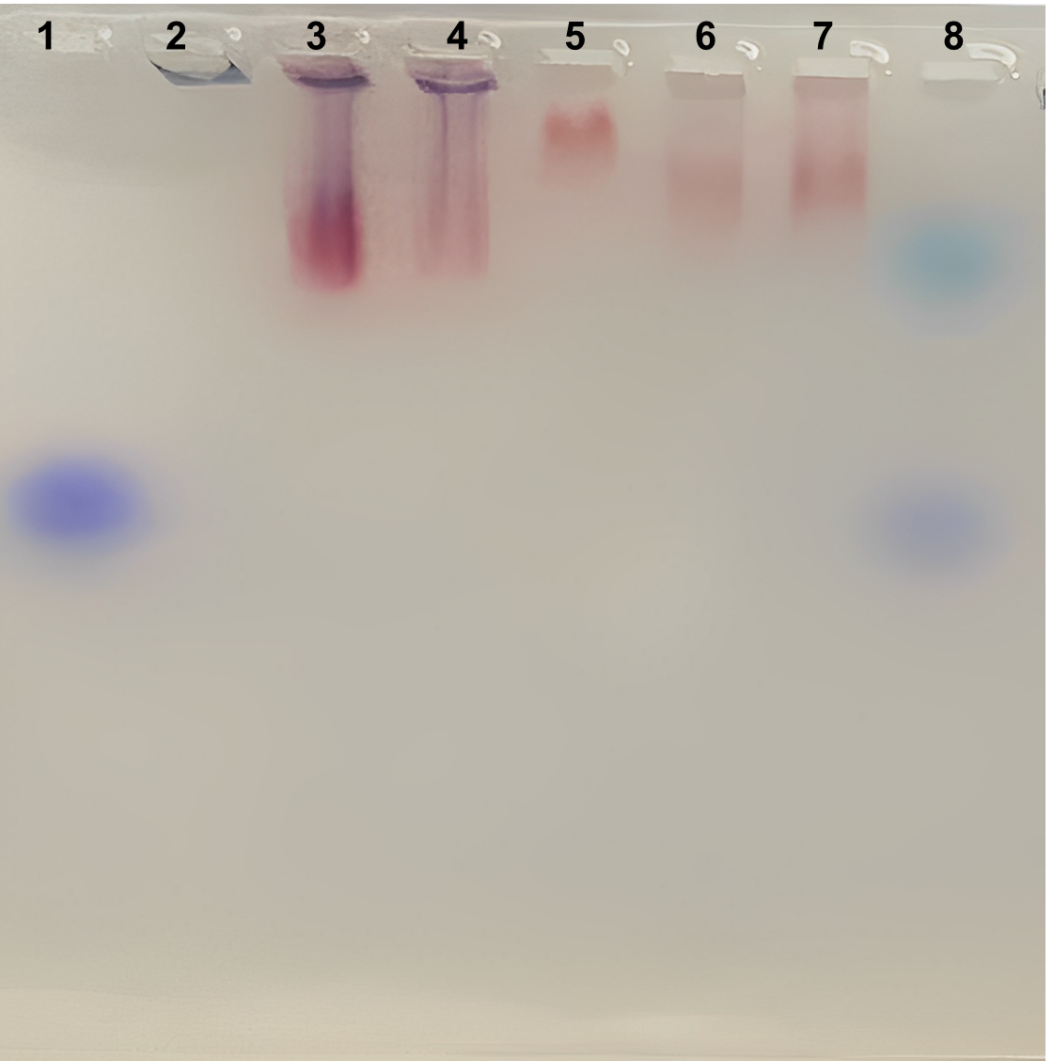
**

**Supplementary Figure 6.** Another exposure of agarose gel analysis of synthetized GNPs and prepared GNPs biosensors. (Lane 1) Marker; (Lane 2) Unfunctionalized citrate- GNPs did not migrate in the gel; (lane 5) PVP- GNPs slightly migrate as a result of PVP capping. (Lane 3 and 4) different concentrations of citrate- GNPs biosensors and (lane 6 and 7) two different concentrations of PVP- GNPs biosensors migration are visible under natural light.


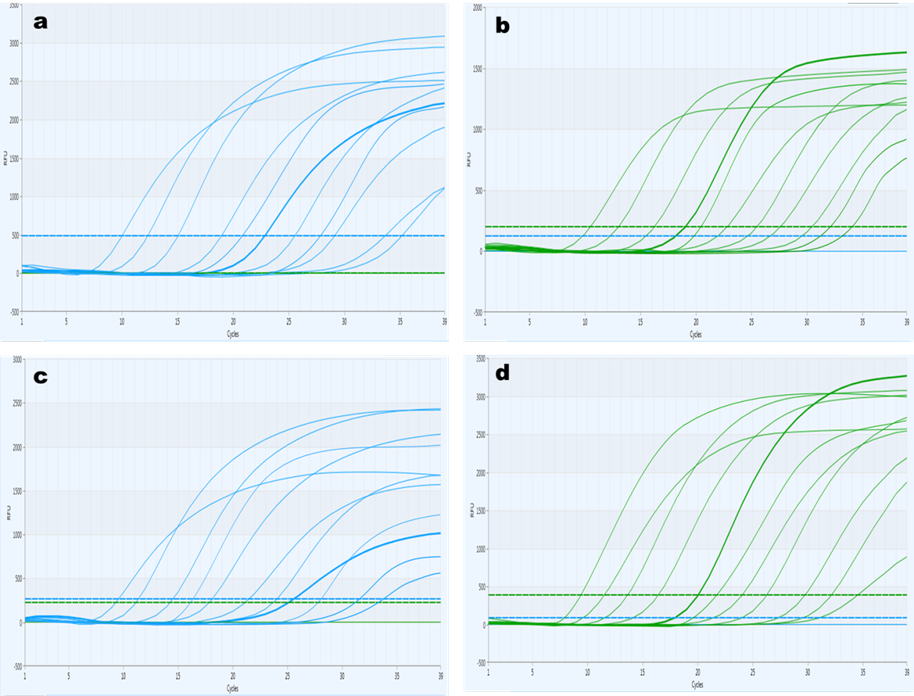


**Supplementary Figure 7.** Amplification curve of EHV-1 and EHV-4 multiplex rPCR assay using GNPs biosensors. (a) Amplification curve of EHV-1 multiplex rPCR assay using EHV-1 and 4 citrate- GNPs biosensor. (b) Amplification curve of EHV-4 multiplex rPCR assay using EHV1 and 4 citrate- GNPs biosensor. (c) Amplification curve of EHV-1 multiplex rPCR assay using EHV1 and 4 PVP- GNPs biosensor. (d) Amplification curve of EHV-4 multiplex rPCR assay using EHV1 and 4 PVP- GNPs biosensor.
